# A Study on Legume-Based Noodles as Staple Food for Office Workers

**DOI:** 10.3389/fnut.2022.807350

**Published:** 2022-03-11

**Authors:** Brian Sumali, Joto Yoshimoto, Hiroto Kobayashi, Mei Yamada, Tetsuya Maeda, Yasue Mitsukura

**Affiliations:** ^1^Faculty of Science and Technology, Keio University, Yokohama, Japan; ^2^Central Research Institute, Mizkan Holdings Co., Ltd., Handa, Japan; ^3^New Business Development, Mizkan Holdings Co., Ltd., Tokyo, Japan

**Keywords:** legume-based noodles, blood glucose level, work performance, *Kansei* engineering, statistical analysis

## Abstract

This study aims to verify the effects of “legume-based noodles” as a staple food for lunch, specifically: blood glucose, cognitive function tests, *Kansei* value, work questionnaires, typing, and body weight. The experiment is divided into two groups: the intervention group (legumes-based noodle) and the control group (regular lunch). Both groups have similar menu except the staple food. The intervention group resulted in a statistically significant lower blood glucose area under the curve (AUC) and lower maximum blood glucose levels during the afternoon work hours on weekdays. In addition, the *Kansei* value “concentration” decreased at the end of the workday in the control group compared to before and after lunch but did not decrease in the intervention group. Furthermore, the number of typing accuracy was higher in the intervention group than in the control group, and the questionnaire responses for “work efficiency” and “motivation” were more positive. These results suggest that eating legume-based noodles may lead to improved performance of office workers.

## Introduction

According to the United Nation's World Population Aging report in 2019, Japan was one of the countries with the most-aged population (34% aged 60 or over), and it is projected to remain so through 2050 (43.9% aged 60 or over) (1). It was also predicted that Japan's workforce will be 40% smaller in 2050, compared to 2000 (2). The long working hours are a major problem in many countries including Japan (3). A study reviewing diseases attributed to long working hours highlighted that: “The Western Pacific, South-East Asia, men, and older people carried higher burdens” (4). Consequently, increasing work efficiency while maintaining the health and well-being of workers in Japan is an important problem to solve.

Along with the aging society comes the health conditions related to old age, such as hearing loss, low vision, back and neck pain, chronic obstructive pulmonary disease (COPD), diabetes, depression, and dementia (5). Diet has been investigated in relation to its ability to promote cognitive function (6–9), and the findings indicate that a healthy diet may have benefits on cognitive function. The cognitive function here refers to one's performance of various mental processes such as memory, learning, reasoning, and language (American Psychological Association).

Additionally, the correlation between sugar and mental concentration was also found in several studies (10–12); although, hyperglycemia is generally not desirable and is linked with the aforementioned diseases (13–15). Furthermore, an unhealthy diet was found to be directly or indirectly correlated with productivity loss (16–18), while the worksite nutrition intervention program was proven effective to improve the wellbeing of the workers (19).

Legumes have been known to produce some health benefits in humans (20–22). They are protein sources with high vitamins, minerals, and antioxidants concentration, and they were also said to be beneficial for patients with diabetes and celiac diseases, as well as for weight management purposes (23). A vegetarian-based diet intervention has also been found to improve work performance, quality-of-life, manage body weight, and cardiovascular diseases (24–26). As such, this study aims to verify the effects of the noodles that are created from legumes, named “legume-based noodles,” as a staple food for lunch, specifically its effects on blood glucose, emotion, work questionnaires, and typing test.

## Materials and Methods

This study was approved by the ethics committees of Keio University and performed in accordance with the Declaration of Helsinki. This study design was registered in UMIN (University hospital Medical Information Network) with registration number UMIN000041239. The study was a non-randomized, open-label, single-arm intervention study. In this section, the foods used in this study and the devices, software, and tools for obtaining the study outcomes are described.

### Foods and Devices

The Memory Performance Index (MPI) score: obtained from the Japanese version of “The MCI Screen” (27, 28) was provided by Millennia Corporation.

Continuous glucose monitoring device: Freestyle Libre from Abbott (29).

*Kansei* values: An emotion analyzer from Dentsu ScienceJam was used (30). Kansei values were computed from the subject's brainwaves. The participants' brainwaves were obtained by an EEG with a sampling rate of 512 Hz (Dentsu ScienceJam Inc.). *Kansei* is originally a Japanese word, and *Kansei* engineering is commonly synonymous with “affective engineering” (American Psychological Association). Originally, *Kansei* engineering meant using a user's subjective feelings to improve a product. In this study, *Kansei* values are the five types of feelings that are converted into numbers. The *Kansei* values are as follows: Stress, Like, Interest, Calmness, and Concentration.

Typing test: C-type software (31) was used for this test. Typical computers were utilized for the typing test, with Windows Operating System and Japanese-layout keyboard (QWERTY JIS).

Questionnaire: The perceived work performance (morning work and afternoon work), motivation (for afternoon work and the day after), and meal contents. The participants were asked to score the questionnaire on a scale of 1–10 points, except for meal contents.

Test foods: White bread (Pasco Shikishima Corporation), Hamburg steak with mixed greens (Seven-Eleven Japan Co., Ltd.), protein drink (Meiji Holdings Co., Ltd.), white rice (Sato Foods Co., Ltd.), salt-grilled mackerel with mixed greens (Seven-Eleven Japan Co., Ltd.), udon noodles (Shidamaya Corporation), simmered flounder with 4 side dishes (Nichirei Corporation), grilled beef (Yoshinoya Co., Ltd.), and legume-based noodles (ZENB Japan Co., Ltd.). The nutrients of these foods are described in Table 1.

**Table 1:** Energy and nutrients from each food used in this study.

	**Energy**	**Protein**	**Fat**	**Carbohydrate**	**Sodium**
	**(kcal)**	**(g)**	**(g)**	**(g)**	**(g)**
White bread	197	5.9	3.1	36.3	0.9
White rice	191	2.7	0	44.1	0
Udon noodles	225	5	1	49.1	1.2
Egg	74	6.29	4.97	0.38	0.07
Legumes-based noodles	261	16.6	2.5	55.1	0.05
Protein drink	75	12.5	0	6.3	0
Hamburg steak	281	17	17.3	16.5	2
Salt-grilled mackerel	336	17.4	29.5	0.2	1
Mixed greens	20	1	0.1	4.5	0
Simmered flounder with	268	15.3	15.1	20	0.705
4 side dishes					
Grilled beef	286	12.6	22.3	8.8	0.8

### Data Acquisition

The experiment was performed for 15 days, with the first week (day 1 to day 7) acting as a control and the second week (day 8 to day 14) as an intervention. Day 15 consists only of MPI measurement, which was conducted before breakfast. The first day of the week (day 1 and day 8) was Saturday, and the last day of the week (day 7 and day 14) was Friday.

The participants were asked to measure their weight every day. The blood glucose level was measured every day, while the *Kansei* score, typing test, and questionnaire were conducted only during working days (Monday–Friday). During Saturdays, only the weight measurement and the MPI test were conducted. The legume-based noodles were boiled in hot water for 6 min just before consumption. Other foods are ready to eat.

**Figure 1:**
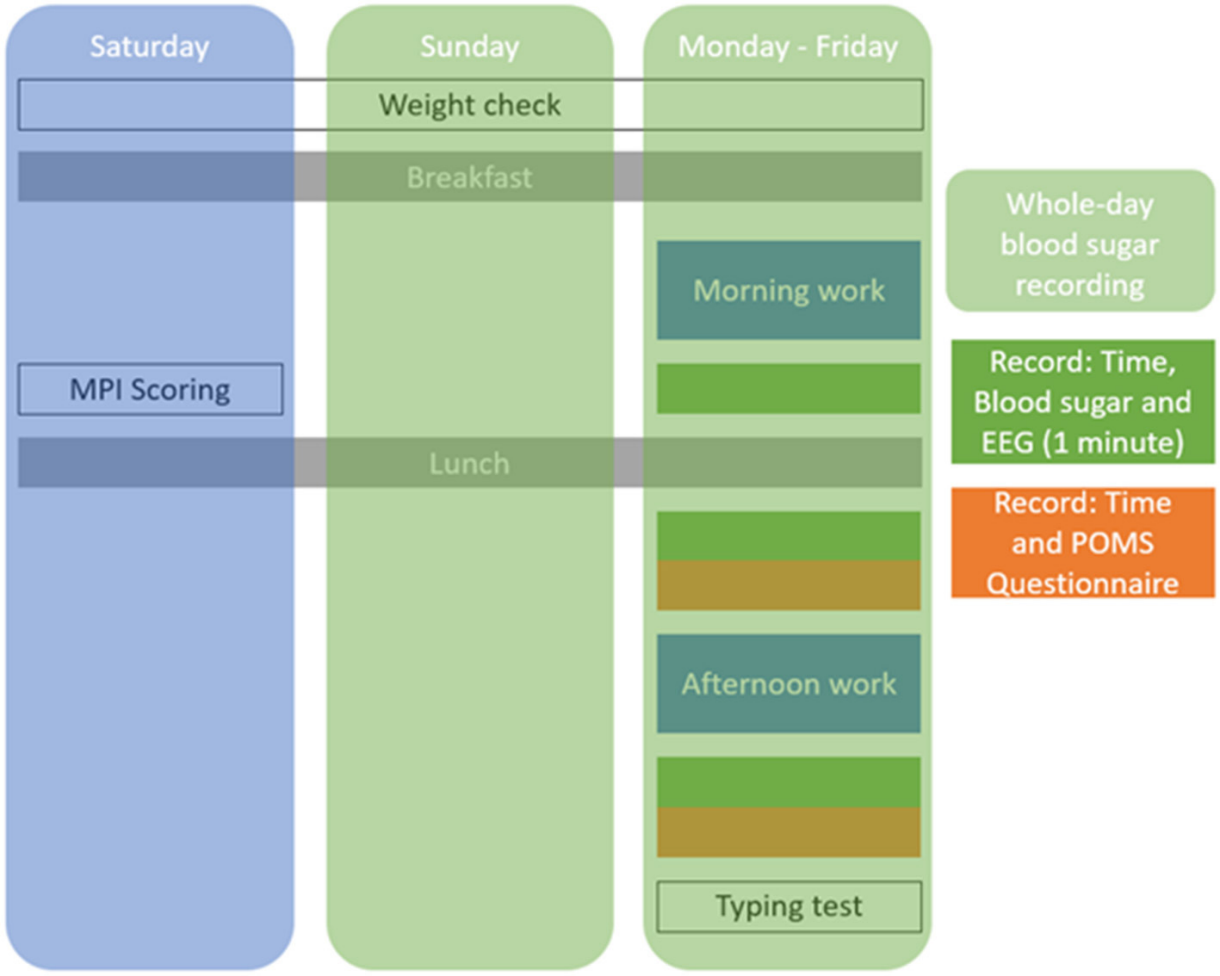
Experimental flow.

During both the control session and the intervention session, the participants' lunch was restricted, as depicted in Table 2. The fat, protein, and carbohydrates in lunch were controlled to be equal. Nevertheless, the time when the participants took their lunch was not regular. The contents and the time of the participant's breakfast were not regular nor restricted. The flowchart of the experiment is described in Figure 1.

**Table 2:** Participants' meal plan during the experiments.

	**Control week**			**Intervention week**		
	**Staple food**	**Dish**	**Drink**	**Staple food**	**Side dish**	**Drink**
Day 1 Sat	White bread	Hamburg steak with mixed greens	Protein milk	Legumes-based noodles	Hamburg steak with mixed greens	Water
Day 2 Sun	Japanese Rice	Salt-grilled mackerel with mixed greens	Protein milk	Legumes-based noodles	Salt-grilled mackerel with mixed greens	Water
Day 3 Mon	Udon noodles with raw eggs	Grilled beef	Protein milk	Legumes-based noodles with raw eggs	Grilled beef	Water
Day 4 Tue	Japanese Rice	Simmered flounder with 4 side dishes	Protein milk	Legumes-based noodles	Simmered flounder with 4 side dishes	Water
Day 5 Wed	Udon noodles with raw eggs	Grilled beef	Protein milk	Legumes-based noodles with raw eggs	Grilled beef	Water
Day 6 Thu	White bread	Hamburg steak	Protein milk	Legumes-based noodles	Hamburg steak	Water
Day 7 Fri	Japanese Rice	Simmered flounder	Protein milk	Legumes-based noodles	Simmered flounder	Water

### Participants

A total of 10 persons (average age = 37 ± 6.43 years; 7 men, 3 women) voluntarily participated in this study and provided written informed consent. Blood glucose level, MPI score, *Kansei* score, Profile of Mood States (POMS) questionnaire, typing test result, and body weight were recorded, as described in Table 3.

**Table 3:** Obtained data from each of the control experiment and intervention experiment.

**Blood glucose level**	**6 days**
	**1 day-off (Sunday) + 5 working days**
**MPI score**	**2 times**
	**first day of the week and last day of the week**
**Kansei score from EEG**	**15 times**
	**3 times per day, 5 working days**
**POMS Questionnaire**	**10 times**
	**2 times per day, 5 working days**
**Typing test result**	**5 times**
	**1 time per day, 5 working days**
**Weight**	**7 times**
	**1 time per day**

The *inclusion criteria* of this study were:

- Healthy men and women aged between 20 and 60;
- Company employees who usually work in an office; and
- Those who understand the contents of the test and provide informed consent.

The *exclusion criteria* of this study were as follows:

- Those with a significant medical history (including receiving medical treatment) or mental illness; and
- Those who are judged by the responsible investigator to be inappropriate for participation in the study.

Of which, 2 *Kansei* scores from the intervention sessions were corrupted, 1 typing test result during the intervention session was lost, and 1 typing test result during the control session was corrupted. These lost and corrupted data were not considered for the analysis. In this article, the weight and the MPI score were not analyzed.

### Statistical Analysis

Statistical analysis was performed using MATLAB and Python in a typical computer. The significance testing was made using a paired *t*-test.

## Results

**Figure 2:**
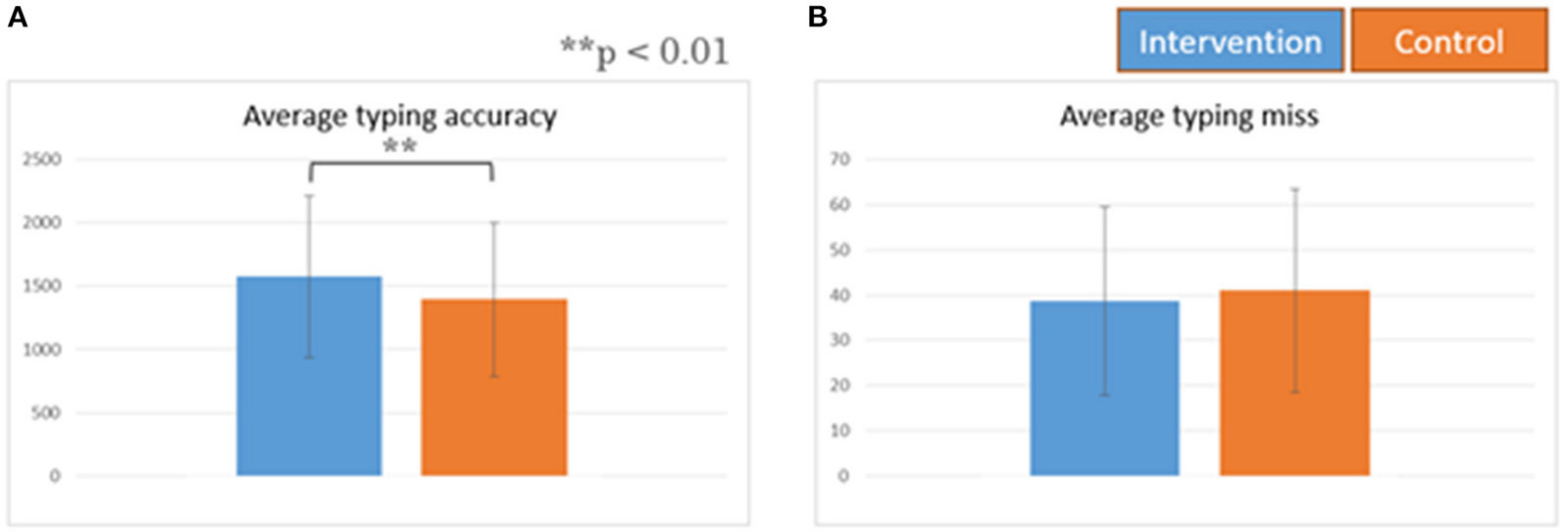
Average typing performance. All data are means ± SD of 10 subjects. **(A)** Average typing accuracy. **(B)** Average typing miss.

**Figure 3:**
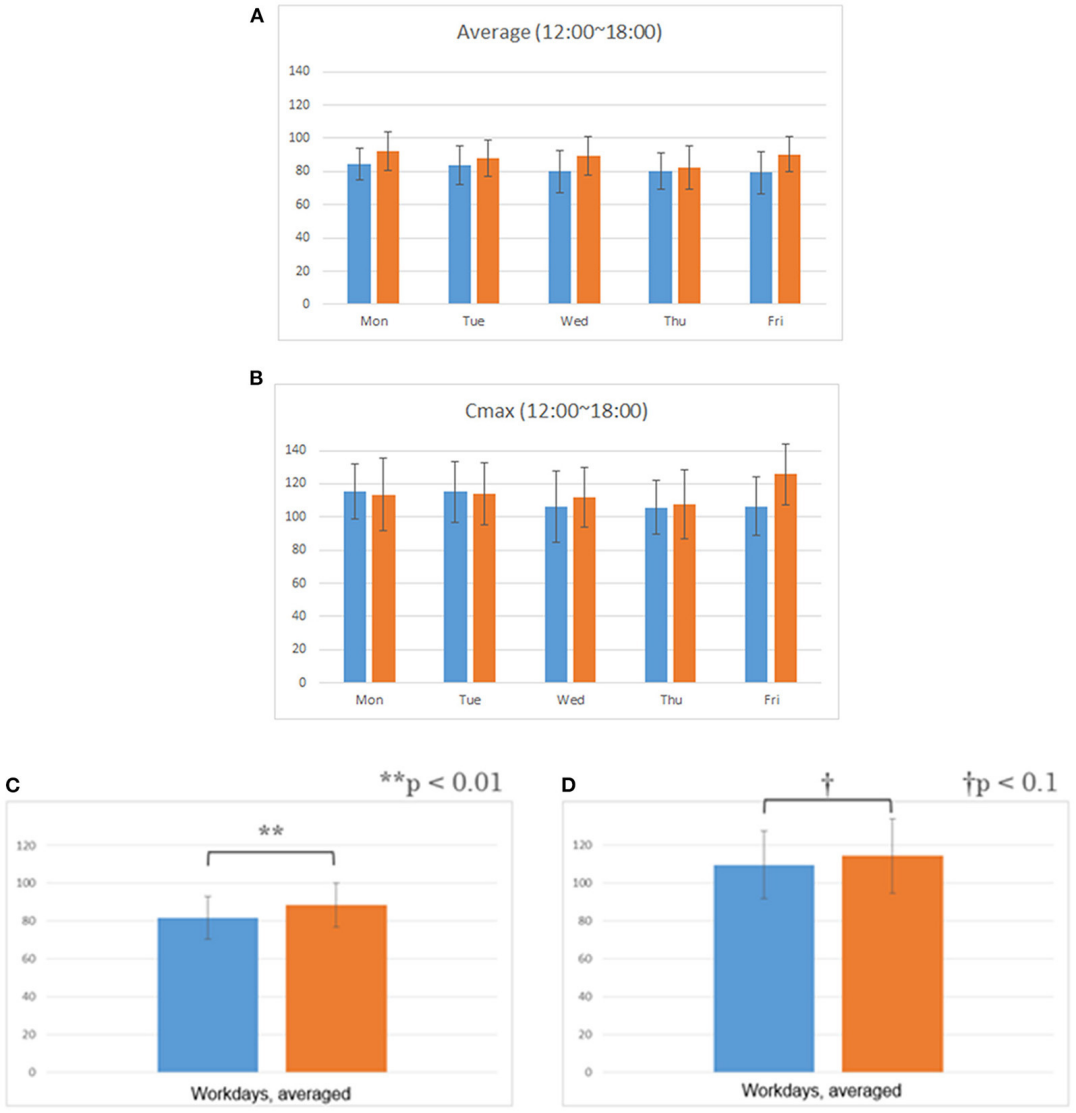
Blood glucose level after lunch. All data are means ± SD of 10 subjects. **(A)** Daily average of blood glucose level. **(B)** Daily average of blood glucose Cmax level. **(C)** Weekly average of blood glucose level. **(D)** Weekly average of blood glucose Cmax level.

**Figure 4:**
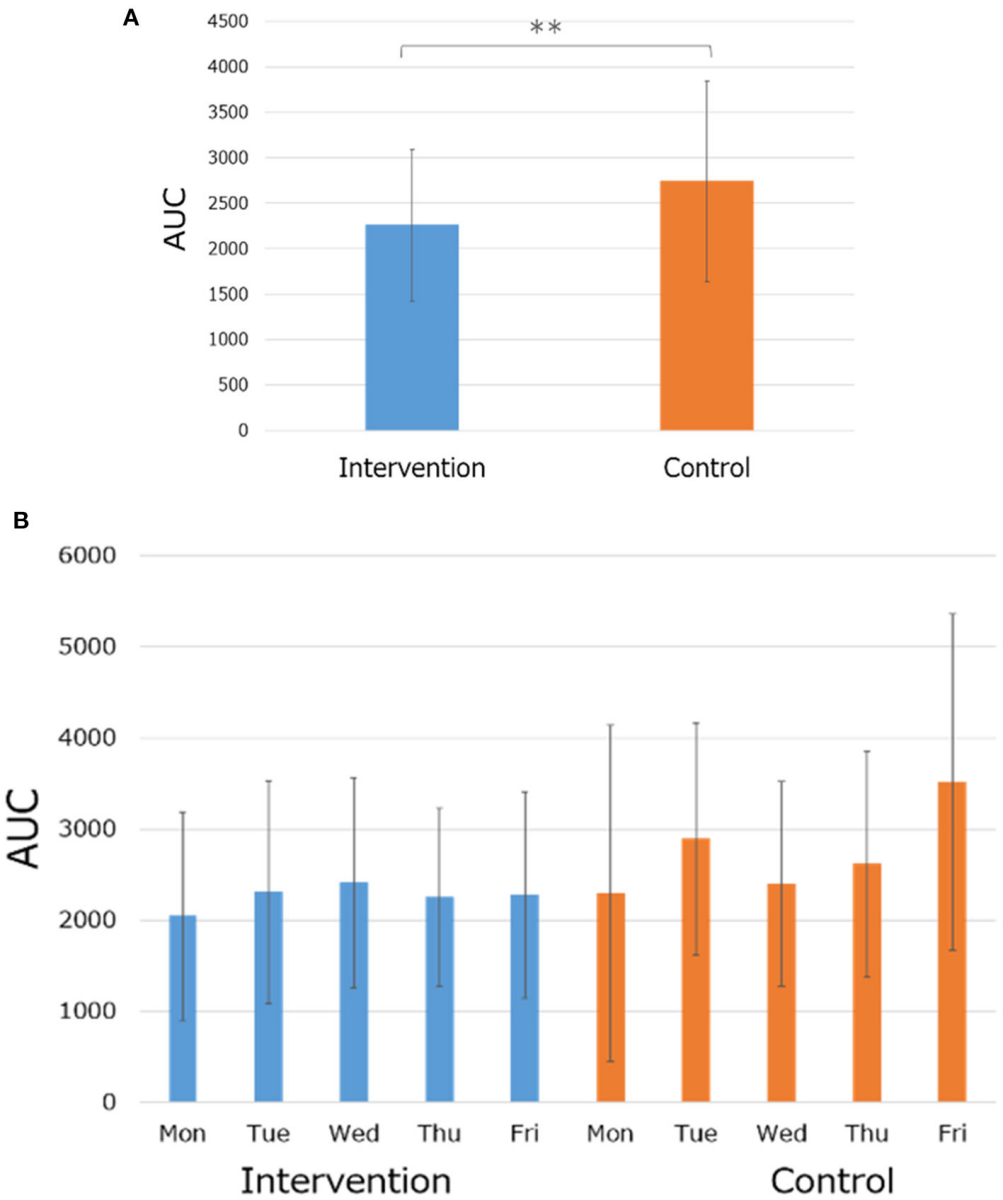
Postprandial blood glucose AUC. All data are means ± SD of 10 subjects. **(A)** Average of five weekdays. **(B)** Data of each weekday.

The consumption of legume-based noodles as lunch resulted in a statistically significant increase in the typing accuracy score, but no difference in the typing miss score (Figure 2). It also resulted in lower blood glucose levels, compared to the control. The comparison of Cmax (highest blood glucose level) also showed similar results: the intervention session results showed a lower Cmax compared to the control session (Figure 3). Similar results were also found in the blood area under the curve (AUC) (Figure 4).

**Figure 5:**
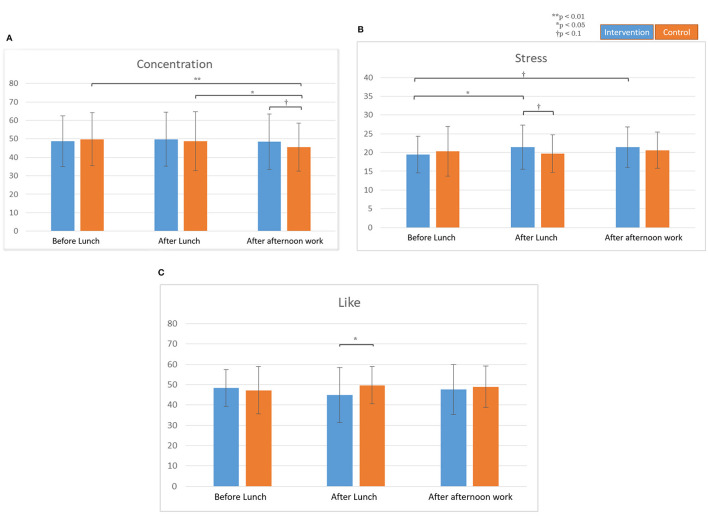
*Kansei* values before and after lunch, and after afternoon work. All data are means ± SD of 10 subjects. **(A)** Concentration values. **(B)** Stress values. **(C)** Like values.

From the results of *Kansei* values, higher concentration values were observed from the subjects during an intervention session (post-afternoon work) compared to the control session (Figure 5A). Higher stress values were observed from the participants during the intervention session (post-meal) compared to the control session. Lower like values were observed from participants during the intervention session (post-meal) compared to the control session (Figures 5B,C).

**Figure 6:**
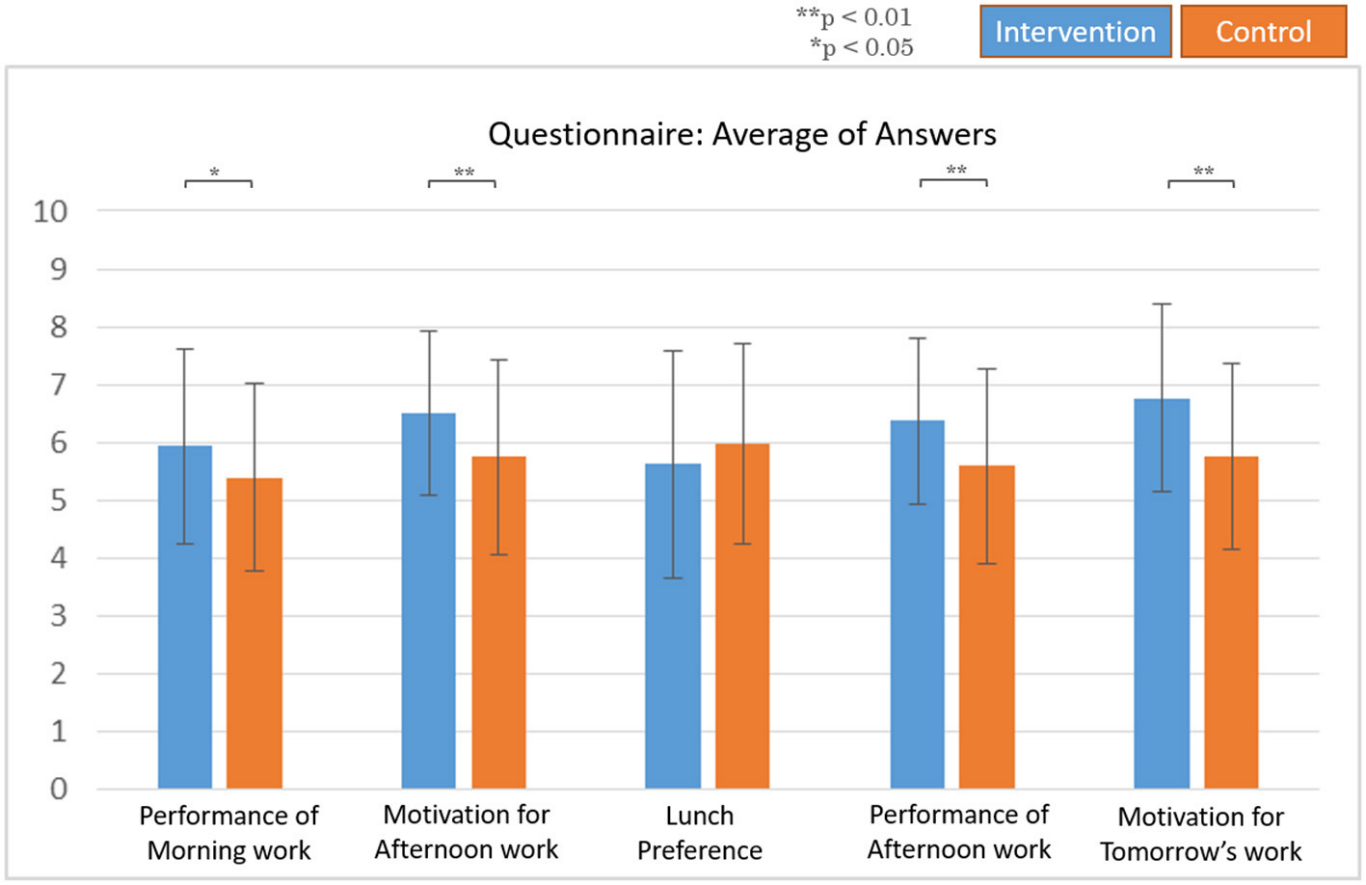
Questionnaire results. All data are means ± SD of 10 subjects.

In the questionnaire, the participants answered that they have better performance during both their morning work and afternoon work and have higher motivation toward the afternoon work and tomorrow's work during the intervention session, compared to the control session (Figure 6).

## Discussion

During the intervention session, the participants were asked to consume legume-based noodles as their staple food for lunch and during the control session. The participants were asked to consume rice, white bread, and Udon noodles as their staple food. Blood glucose levels, *Kansei* values from EEG, POMS questionnaire, and typing test scores were obtained from both sessions. Statistical analysis was made to compare the values obtained from the intervention session and the control session.

From the results of the afternoon work, the intervention session showed a statistically significant lower average blood glucose level, blood glucose AUC, and blood glucose Cmax compared to the control session. The legume-based noodles used in this study were proven to be low GI in a previous study, so these results are consistent with it (32).

It is generally believed that average blood glucose level, blood glucose AUC, and blood glucose Cmax increase after meals (33). In particular, it is considered that the increase is even higher in a diet that is high in sugar such as white rice (34).

Not increasing the average blood glucose level, blood glucose AUC, and blood glucose Cmax after meals reduces the burden on the body and is good for the health (35–40).

In this study, the intervention session showed a significantly higher typing score at the end of the workday than the control group. This may be related to the gradual increase in blood glucose when the subject ingested the test food at lunch. Eating low glycemic index (GI) foods has positive effects on cognitive performance, and it is possible that the same thing happened in this study (41, 42).

Additionally, while the subjects from the control session had a significantly lower average concentration at the end of their afternoon work compared to their average concentration during pre-lunch and post-lunch, the reversed phenomena were found during the intervention session; the average concentration values at the end of the afternoon work were significantly higher compared to the pre-lunch and post-lunch values.

Both typing tests and questionnaires were utilized to measure the outcomes related to work. The typing accuracy, as quantified work performance along with the questionnaire's motivation and work performance, were compared, and in all cases, the intervention session showed better results.

From the results of this study, it was shown that legume-based noodles may improve short-term work performance and motivation, so, we believe that a long-term intake is expected to improve cognitive ability and prevent dementia.

However, some limitations of this study must be considered when interpreting the results of this study. First, the number of study subjects is relatively small. Second, as this study is performed in Japan, most of the ethnicity of the subjects are Asian. Third, the subjects of this study consumed their meal based on their self-control; this might lead to more variance compared to a more restrictive experiment.

In an age where people are expected to live longer and healthier lives, we believe it is highly worthwhile to research foods that are less likely to raise blood glucose levels and have the potential to improve health and/or work performance.

## Conclusions

This study aimed to verify the effects of “legume-based noodles.” Several measures were taken in this study and as the outcome, the intervention group resulted in statistically significant lower blood glucose for AUC, lower maximum blood glucose levels, and preventing the decrease of “concentration” aspect in *kansei*. Furthermore, the typing accuracy was better in the intervention group than in the control group, and the questionnaire responses for “work efficiency” and “motivation” were more positive. This study found that the consumption of legume-based noodles improves the work performance of office workers, with several objective outcomes. As future work, more participants, both in number, age group, and ethnicity, are needed. Additionally, less variability in the control and intervention group may lead to more specific results. Analysis of the weight and the MPI scores might also yield interesting results.

## Data Availability Statement

The raw data supporting the conclusions of this article will be made available by the authors, without undue reservation.

## Ethics Statement

The studies involving human participants were reviewed and approved by Keio University Bioethics Committee. The patients/participants provided their written informed consent to participate in this study.

## Author Contributions

BS, JY, HK, MY, TM, and YM: conceptualization, writing, reviewing, and editing. BS and YM: methodology, validation, formal analysis, and writing the original draft preparation. BS: software. YM: investigation, data curation, supervision, project administration, and funding acquisition. JY, HK, MY, and TM: resources and visualization. All authors contributed to the article and approved the submitted version.

## Conflict of Interest

JY, HK, MY, and TM are employees of Mizkan Holdings, Co., Ltd. None of the principal investigators involved in this study or their family members are shareholders in Mizkan Holdings Co., Ltd., or are company officers, directors, or advisors. The remaining authors declare that the research was conducted in the absence of any commercial or financial relationships that could be construed as a potential conflict of interest.

## Publisher's Note

All claims expressed in this article are solely those of the authors and do not necessarily represent those of their affiliated organizations, or those of the publisher, the editors and the reviewers. Any product that may be evaluated in this article, or claim that may be made by its manufacturer, is not guaranteed or endorsed by the publisher.
